# Storage damage of cold-stored whole blood in CPDA maintenance fluid during the whole effective storage period

**DOI:** 10.3389/fcell.2025.1610009

**Published:** 2025-08-22

**Authors:** Yingyu He, Yiquan Zhang, Wanbing Liu, Lidong Zhang, Yingkai Xu, Zihan Yuan, Junying Li, Lei Liu, Fangxiong Cheng

**Affiliations:** ^1^ Department of Transfusion, Wuhan Fourth Hospital, Wuhan, Hubei, China; ^2^ Department of Clinical Laboratory, Nantong Third People’s Hospital, Affiliated Nantong Hospital 3 of Nantong University, Nantong, Jiangsu, China; ^3^ Department of Transfusion Medicine, General Hospital of Central Theater Command, Wuhan, Hubei, China; ^4^ Medical College, Wuhan University of Science and Technology, Wuhan, Hubei, China

**Keywords:** whole blood, storage damage, hemostatic resuscitation, transfusion, trauma

## Abstract

**Background:**

Massive hemorrhage is a leading cause of mortality among trauma patients. To date, whole blood (WB) remains the preferred resuscitation fluid on the battlefield and in pre-hospital emergency care. However, components of WB inevitably undergo storage-related damage, and differences in the duration of storage may lead to varying clinical outcomes after transfusion. This study will involve monitoring cold-stored whole blood (CS-WB) to assess variations in the concentration and activity of each component during *in vitro* storage.

**Methods:**

20 bags of WB (400 mL each) from healthy donors were stored at (4 ± 2) °C. Aliquots were collected at storage days 0, 7, 14, 21, 28, and 35 for analysis. On each testing day, storage lesion related indicators of red blood cells (RBCs), plasma components, and platelets were respectively detected.

**Results:**

On the 14th day of CS-WB storage, no significant changes were found in the morphology, quantity, and function of RBCs. The oxygen carrying capacity of RBCs slightly decreased. Moreover, CS-WB was able to maintain good coagulation function, platelets morphology and hemostatic activity. On the 21st day of CS-WB storage, the oxygen carrying capacity and ATP content of RBCs showed a more significant decrease. Platelets showed characteristic ultrastructural damage and progressive decline in hemostatic function. However, thrombelastogram (TEG) results showed CS-WB could still maintain a certain level of coagulation function and thrombotic ability. By the day 28 of storage, coagulation activity decreased alongside elevated hemolysis markers, indicating progressive and remarkable storage lesion development. On the 28th day of storage, the coagulation activity significantly decreased with the increase of hemolysis markers, indicating that storage damage to active components such as RBCs, platelets, and coagulation factors in CS-WB was ongoing and remarkably developing.

**Conclusion:**

These findings show that CS-WB maintained within 14 days of storage provides optimal preservation of critical hemostatic properties, including RBCs oxygen-carrying capacity, coagulation factors function, and platelets hemostatic performance. This storage window holds particular clinical relevance for hemorrhagic shock resuscitation in resource-constrained scenarios, such as military medicine or prehospital trauma care systems.

## 1 Introduction

Trauma remains the main reason of disability and death of young persons all over the world despite continuing advances in resuscitation, critical care, and trauma surgery. Hemorrhagic shock is a leading cause of mortality among patients with severe trauma. Timely transfusion of blood products is critical for buying time for surgical interventions to stop bleeding and preventing organ failure and death in both military and civilian trauma settings. The current blood transfusion strategy for trauma patients is the balanced administration of equal numbers of units of blood components in 1:1:1 fashion (RBCs to plasma to platelets). However, administration of components in the manner of reconstituted whole blood is demonstrated to be associated with resuscitation-associated changes including anemia, thrombocytopenia, and coagulation factor dilution (Murdock et al., 2014). Cold-stored (2 °C–6 °C) whole blood (CS-WB) has gained increasing attention as a resuscitation option for hemorrhagic shock (Sperry et al., 2023; van der Horst et al., 2023), which provides RBCs, plasma, and platelets simultaneously in a single unit. CS-WB can rapidly restore blood volume, enhance tissue oxygenation and improve coagulation dysfunction at the same time.

The extensive military experience has shown that use of CS-WB for the resuscitation of patients with traumatic hemorrhage is feasible (Almog et al., 2024; Shea et al., 2024; Torres et al., 2024). Supporting these findings, a recent German Bundeswehr review emphasized that low-titer O whole blood offers clear medical and logistical benefits for austere operational settings, despite ongoing regulatory and cold-chain management challenges (Sauer and Meyer, 2025). Clinical evidence further substantiates these observations: a pilot randomized controlled trial reported reduced early transfusion requirements without compromising survival (Cotton et al., 2013); a forward surgical cohort study observed a nearly ten-fold decrease in adjusted mortality with fresh whole blood (Nessen et al., 2013); and a large-scale TQIP analysis confirmed that whole blood transfusion significantly shortened ICU stays and reduced major complications compared to balanced component therapy, while maintaining equivalent overall survival rates (Campbell et al., 2025). Collectively, these findings suggest that CS-WB enhances hemostatic efficacy and logistical simplicity, rendering it particularly valuable for battlefield or other resource-limited environments.

Despite these advantages, CS-WB is approved storage period ranges from 21 to 35 days based on the anticoagulant utilized and experiences a series of biological changes during storage. These changes, often called “storage damage,” may compromise its function, efficacy, and resuscitation effect in severely hemorrhagic patients. The previous research indicated that prolonged storage of CS-WB can lead to RBC hemolysis, decreased platelet counts, and diminished coagulation factor activity (Bjerkvig et al., 2020). Although a few studies have documented these alterations in CS-WB up to 14 or 21 days during cold storage, the efficacy of CS-WB beyond these time points remains insufficiently characterized. Furthermore, there is still a lack of systematic and comprehensive research regarding the change of various components (e.g., RBCs, platelets, and key coagulation factors) with the extension of storage time and the possible impact of these changes on the transfusion effect. A better understanding of this question may shed insights into potential mechanisms related to safety and therapeutic efficacy by which WB differs from blood component transfusion for trauma patients.

The present study aims to systematically investigate the storage-lesion of CS-WB components including RBC oxygen-carrying capacity, platelet ultrastructure, and coagulation factor levels over multiple storage intervals. By clarifying how storage-lesion affects the structure, concentration, and function of these active ingredients in CS-WB, we seek to provide laboratory evidence on the optimal use strategy of CS-WB for resuscitating trauma patients with hemorrhagic shock.

## 2 Materials and methods

### 2.1 WB collection and sample preparation

Twenty healthy male donors of 18 years of age and older (age range from 21 to 34 years) provided informed consent for two units WB donation. Fifteen of these donors had group B blood, and five of them had group AB blood. Two units of 400 mL of WB were collected in a citrate phosphate dextrose adenine (CPDA) bag composed of polyvinyl chloride material (Shuangwei, Nanjing, China) from each donor according to an approved institutional standard operating procedure. All WB units were placed on open-work perforated racks at 4 °C with the label side facing downward, leaving the gas-permeable rear film fully exposed. These 20 bags of WB products were stored unagitated in a blood bank refrigerator set to 4 °C for 35 days after passing the blood quality test (Sivertsen et al., 2018).

Storage-lesion testing was performed on days 0, 7, 14, 21, 28, and 35 from the day of blood collection (Day 0, baseline; room temperature). On each testing day, the blood bags were mixed via gentle inversion to ensure thorough mixing prior to sampling. A 16 mL aliquot of WB was obtained using a sterile 20 mL syringe. From this aliquot, 2 mL of each sample was transferred into a centrifuge tube and spun at 3,000 rpm for 10 min. The separated plasma was collected and stored at −40 °C until further analysis. The remaining 14 mL of the sample was used for subsequent measurements, including RBCs, plasma, and platelet damage analyses. From days 0–35, a total of 96 mL of blood were removed from each collection bag, leaving 304 mL leftover in each bag at the conclusion of this study (Jobes et al., 2011). According to our institutional quality-control protocol (minimum residual volume ≥300 mL), all units in this study retained >300 mL after serial sampling. At each sampling time point, a complete blood count was performed using a Sysmex XS-1000i hematology analyzer (Sysmex, Japan). The coagulation kinetics of CS-WB was conducted with Haemonetics 5000 Thromboelastograph (TEG, Haemonetics, America) using corollary reagents with kaolin activation. The relevant parameters such as R (min), K (min), angle (°), and MA (mm) were determined.

### 2.2 RBCs storage damage assessment

RBCs morphological changes were observed using a Nanjing Jiangnan Yongxin Optics BM2000 optical microscope (Nanjing Jiangnan Yongxin Optics, China) to detect potential shape alterations. The osmotic fragility test was performed by exposing RBCs samples to sodium chloride (NaCl) solutions at varying concentrations. After gentle mixing and centrifugation (3,600 rpm, 10 min), the absorbance at 540 nm of the supernatant was measured to assess RBC susceptibility to hypotonic stress. The hemolysis increment was calculated based on the degree of RBC hemolysis in 0.56% NaCl solution, which was then used to derive the EC50 value (Pulliam et al., 2020). RBCs intracellular ATP content was determined by Enzyme-Linked Immunosorbent Assay (ELISA). Each sample was mixed with ddH_2_O at a 1:4 ratio and boiled for 10 min to release ATP. The supernatant was then analyzed using an ATP detection kit (Sigma, America) following the manufacturer’s instructions. The detection result was calculated as μmol/g Hb. The measurement process of RBCs membrane integrity and apoptotic status was conducted with Aisen Gene D2040 Flow cytometry (Aisen Gene, China) using corollary reagents. Eosin-5′-maleimide (Sigma, America) was used to evaluate RBCs membrane integrity, while Annexin V (BD Biosciences, America) binding indicated phosphatidylserine (PS) exposure on apoptotic RBCs. The relevant results were expressed as percent positivity. The free hemoglobin (FHb) content was determined by detecting plasma samples thawed at room temperature using plasma FHb assay kit (Beijing Ruierda Biotechnology, China) and following the manufacturer’s instructions. The 2,3-diphosphoglycerate (2,3-DPG) content was determined and analyzed using a human-2,3-DPG ELISA kit (Tianjin Anoric Biotechnology, China) following the manufacturer’s instructions.

### 2.3 Plasma functions and metabolic changes assessment

At each sampling time point, routine coagulation function were measured using a Werfen ACL TOP coagulometer (Werfen, Spain) and corollary reagents (Instrumentation Laboratory, America). These parameters included activated partial thromboplastin time (APTT), prothrombin time (PT), thrombin time (TT), fibrinogen (Fib), and coagulation factor VIII (FVIII) activity. Simultaneously, biochemical routine parameters were measured using a Beckman DxC 800 Pro biochemical analyzer (Beckman, America) and corollary reagents (Beckman, America). These data included albumin (ALB), globulin (GLB), carbon dioxide (CO_2_), lactate (Lac), potassium ion (K^+^), sodium ion (Na^+^), calcium ion (Ca^2+^), glucose (GLU), and lactic dehydrogenase (LDH). The key coagulation factor CF10 and CF13 were determined using human CF10 and CF13 ELISA kit (Tianjin Anoric Biotechnology, China) following the manufacturer’s instructions. All assays were performed in triplicate to ensure accuracy and reproducibility. Plasma samples were diluted or processed according to the analyzer’s operational guidelines.

### 2.4 Platelets storage damage assessment

The measurement process of platelet activation and apoptotic status was conducted with Aisen Gene D2040 Flow cytometry (Aisen Gene, China) using corollary reagents. CD62P (BD Biosciences, America) was used to evaluate platelet activation, while Annexin V (BD Biosciences, America) binding indicated PS exposure on apoptotic platelets. The flow cytometry results were expressed as percent positivity. Platelets morphological changes were observed using Hitachi SU8020 scanning electron microscopy (SEM) (Hitachi, Japan) and Hitachi HT7700 transmission electron microscopy (TEM) (Hitachi, Japan) to detect external morphological features and internal structural alterations, respectively.

### 2.5 Statistical analysis

All data were processed using Statistical Package for the Social Sciences (SPSS) version 26.0 software, and graphs were generated with GraphPad Prism 9. Measurement data were presented as the means and standard deviations (SD). The normality assumption was tested using the Shapiro–Wilk test. Homogeneity of variances was assessed using both the Bartlett’s test and the Brown–Forsythe test. One-way analysis of variance (ANOVA) was employed to compare differences among multiple groups, treating the first group as the control. Post hoc multiple comparisons were performed using the Sidak method. For global error-rate control across 190 planned contrasts, raw P values were additionally corrected by the Benjamini–Hochberg procedure (Supplementary Table S1). Means between groups were compared sequentially, and a two-sided α of less than 0.05 was considered statistically significant.

*A priori* power analysis was performed in PASS (one-way repeated-measures ANOVA, α = 0.05, power = 0.90). Specifying a medium within-subject effect size (Cohen f = 0.30) across six time-points indicated that 18 bags of WB would be sufficient. To allow for potential exclusions, 20 bags were finally enrolled.

## 3 Results

### 3.1 RBC storage damage in CS-WB

**FIGURE 1:**
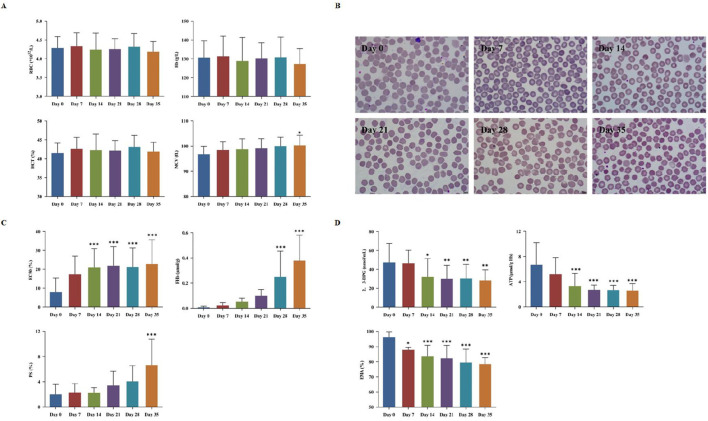
Red blood cells (RBCs) storage damage in cold-stored whole blood (CS-WB). **(A)** Blood routine indicators. **(B)** RBCs morphological changes under a microscope (100×). Day 0: normal and intact RBCs; Day 7: few small spur-like projections; Day 14: slight increase in spur-like projections; Day 21: notable increase in spur-like projections; Day 28: extensive spiculated RBCs with prominent spur formation; Day 35: majority of RBCs exhibited significant spiculated features. **(C)** Destruction of RBCs; **(D)** Changes in RBCs oxygen-carrying capacity and membrane proteins. WB samples were collected from 20 bags of CS-WB stored at 2 °C–6 °C every 7 days. Day 0 was used as the control group. Data are presented as the mean ± SD for each group. Bar graphs represent the mean values, with error bars indicating SD. *P < 0.05, **P < 0.01, ***P < 0.001 indicate statistically significant differences compared to the control group.

The detection indicators of the day 0 samples were used as the controls. The RBCs count, hematocrit (Hct), and hemoglobin (Hb) content showed no significant changes throughout the 35 days observation period, while the mean corpuscular volume (MCV) showed significantly increase after 35 days of storage (Table 1; Figure 1A). The microscopic observation results indicated spikes began to appear on the surface of a small amount of RBCs on the 7th day of storage. Moreover, the proportion of spike RBCs was increasing with the prolongation of storage time. By day 35, most of all RBCs had prominent spines, and some normal RBCs showed significant morphological changes (Figure 1B). Meanwhile, the levels of FHb and PS both rose continuously, respectively exhibiting a significant increase by day 28 and 35 (*P* < 0.05). By this time, the average FHb level reached 0.25 ± 0.2 μmol/L, a level still well below internationally accepted haemolysis limits for transfusable blood, and PS was 2.3 times higher than at the beginning of storage (Figure 1C). Additionally, both 2,3-DPG and ATP declined over the course of CS-WB storage (Figure 1D). On the 14th day of storage, both these two parameters had decreased significantly relative to day 0 (*P* < 0.05). Notably, ATP content dropped rapidly during the second week, reaching 50.18% below its initial level by day 14.

**TABLE 1:** Trends in blood routines during the storage period of whole blood.

Blood routines	Day 0	Day 7	Day 14	Day 21	Day 28	Day 35
WBC (*10^9^/L)	5.33 ± 0.84	4.55 ± 0.81	3.98 ± 1.17*	3.60 ± 0.80*	3.30 ± 0.85*	3.17 ± 0.85*
RBC (*10^12^/L)	4.28 ± 0.29	4.33 ± 0.35	4.24 ± 0.44	4.27 ± 0.27	4.32 ± 0.35	4.18 ± 0.26
Hb (g/L)	130.70 ± 8.83	131.40 ± 10.64	128.90 ± 12.46	130.30 ± 8.27	130.8 ± 10.69	127.20 ± 8.33
MCV (fL)	96.77 ± 3.19	98.50 ± 3.29	98.78 ± 4.07	99.12 ± 3.77	100.00 ± 3.60	100.30 ± 4.05*
HCT (%)	41.47 ± 2.69	42.62 ± 3.09	42.31 ± 4.21	42.20 ± 2.61	43.15 ± 3.06	41.94 ± 2.44
MCH (pg)	30.49 ± 0.92	30.35 ± 0.84	30.28 ± 0.97	30.60 ± 0.93	30.30 ± 0.97	30.40 ± 0.92
MCHC (g/L)	315.30 ± 9.45	308.20 ± 8.15	307.00 ± 11.24*	308.90 ± 7.60	303.10 ± 8.39*	303.30 ± 10.56*

* Indicates a statistically significant difference compared to the Day 0 group.

WBC, white blood cell count; RBC, red blood cell count; Hb = Hemoglobin; MCV, mean corpuscular volume; HCT, hematocrit; MCH, mean corpuscular hemoglobin; MCHC, mean corpuscular hemoglobin concentration.

### 3.2 Plasma components storage damage in CS-WB

As shown in Table 2, a series of biochemical indicators in plasma were measured during the entire effective storage period of CS-WB. During the storage period, the levels of Lac, K^+^, and LDH gradually increased, while the levels of Na^+^, CO_2_, and GLU evidently decreased with the extension of storage time. In addition, the levels of ALB and GLB had no significant changes. According to the national standard related to WB product (GB18469-2012), all these indicators were within the normal range.

**TABLE 2:** Changes of biochemical indicators during whole blood storage period.

Biochemical indicators	Day 0	Day 7	Day 14	Day 21	Day 28	Day 35
ALB (g/L)	38.62 ± 1.69	38.85 ± 1.60	38.64 ± 1.39	38.85 ± 1.55	38.43 ± 1.54	39.94 ± 1.38
GLB (g/L)	23.76 ± 3.21	24.58 ± 2.28	24.58 ± 1.96	24.79 ± 2.34	24.77 ± 2.48	24.68 ± 2.77
CO_2_ (mmol/L)	17.21 ± 0.99	15.82 ± 1.01	13.87 ± 2.02*	12.28 ± 1.62*	10.81 ± 1.98*	7.87 ± 2.57*
Lac (mmol/L)	2.65 ± 0.06	5.06 ± 0.75*	6.54 ± 1.21*	7.87 ± 0.96*	9.78 ± 0.92*	9.59 ± 0.60*
K^+^ (mmol/L)	4.41 ± 0.55	10.92 ± 1.42*	14.13 ± 1.40*	19.51 ± 2.24*	23.58 ± 3.04*	25.65 ± 2.22*
Na^+^ (mmol/L)	169.30 ± 2.23	165.80 ± 2.88*	164.50 ± 3.05*	160.20 ± 2.17*	155.90 ± 2.39*	155.9 ± 2.59*
Ca^2+^ (mmol/L)	1.25 ± 0.09	1.21 ± 0.04	1.28 ± 0.08	1.20 ± 0.08	1.20 ± 0.04	1.57 ± 0.23*
GLU (mmol/L)	32.81 ± 5.71	29.62 ± 6.15	26.98 ± 6.90	25.18 ± 7.35*	22.40 ± 7.57*	21.84 ± 8.01*
LDH (U/L)	181.70 ± 32.24	246.70 ± 96.59	319.50 ± 130.40*	437.60 ± 144.60*	547.8 ± 147.00*	704.80 ± 170.30*

* Indicates a statistically significant difference compared to the Day 0 group.

ALB, albumin; GLB, globulin; Lac = Lactic Acid; K^+^ = potassium; Na^+^ = Sodium; Ca^2+^ = Calcium; GLU, glucose; LDH, lactate dehydrogenase.

**FIGURE 2:**
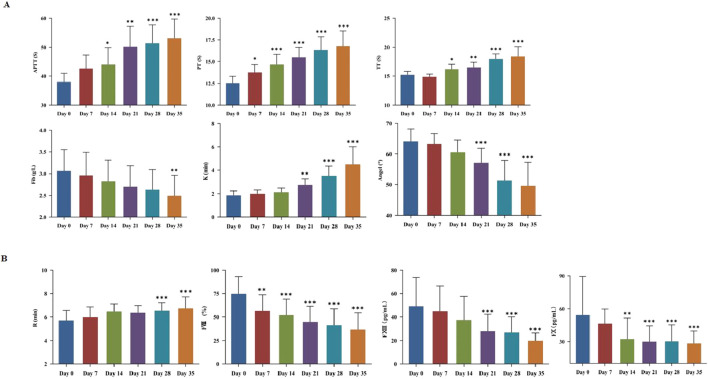
Plasma storage damage in cold-stored whole blood (CS-WB). **(A)** Coagulation function; **(B)** Evaluation of coagulation factors. WB samples were collected from 20 bags of CS-WB stored at 2 °C–6 °C every 7 days. Day 0 was used as the control group. Data are presented as the mean ± SD for each group. Bar graphs represent the mean values, with error bars indicating SD. *P < 0.05, **P < 0.01, ***P < 0.001 indicate statistically significant differences compared to the control group.

The coagulation function was evaluated by TEG testing and traditional blood coagulation test (Supplementary Table S2). The relevant parameters likewise exhibited time-dependent alterations. APTT and PT were both prolonged with increased storage time (Figure 2A). Notably, PT value was extended from (12.54 ± 0.77) s to (13.76 ± 0.93) s (*P* < 0.05) on day 7. On day 14, APTT was increased from (38.04 ± 2.9) s to (44.10 ± 5.79) s (*P* < 0.05), and TT was prolonged from (15.21 ± 0.60) s to (16.20 ± 0.90) s (*P* < 0.05). On day 21, TEG-K value was increased from (1.87 ± 0.36) min to (2.74 ± 0.53) min (*P* < 0.05), while TEG-Angle value was decreased from (64.15 ± 3.96)° to (57.13 ± 4.71)° (*P* < 0.05). On day 35, Fib value was decreased from (3.06 ± 0.49) g/L to (2.50 ± 0.47) g/L (*P* < 0.05). As shown in Figure 2B, TEG-R value was significantly prolonged on day 28 compared to day 0 (*P* < 0.05). Meanwhile, the levels of key coagulation factor such as FⅧ, FⅩ, and FⅩⅢ in plasma were individually determined. Specifically, FⅧ activity was decreased by 24.33% on day 7. FⅩ content was dropped by 40.40% on day 14, and FⅩⅢ content was reduced by 43.29% on day 21.

### 3.3 Platelets storage damage in CS-WB

**FIGURE 3:**
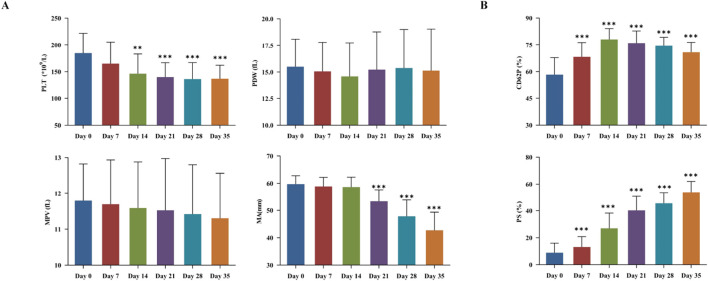
Platelet storage damage in cold-stored whole blood (CS-WB). **(A)** Platelets number and function; **(B)** Platelets activation and apoptosis. WB samples were collected from 20 bags of CS-WB stored at 2 °C–6 °C every 7 days. Day 0 was used as the control group. Data are presented as the mean ± SD for each group. Bar graphs represent the mean values, with error bars indicating SD. *P < 0.05, **P < 0.01, ***P < 0.001 indicate statistically significant differences compared to the control group.

During refrigerated storage, mean platelet volume (MPV) and platelet distribution width (PDW) were largely unchanged, while platelet count and function were declined over time. On day 21, a statistically significant reduction in platelet count was observed (Figure 3A) (*P* < 0.05). The expression levels of PS and CD62P on the platelet membrane surface were increased over time. Significant differences were both observed on day 7 (*P* < 0.05). Notably, PS expression had nearly doubled on day 14 (Figure 3B).

**FIGURE 4:**
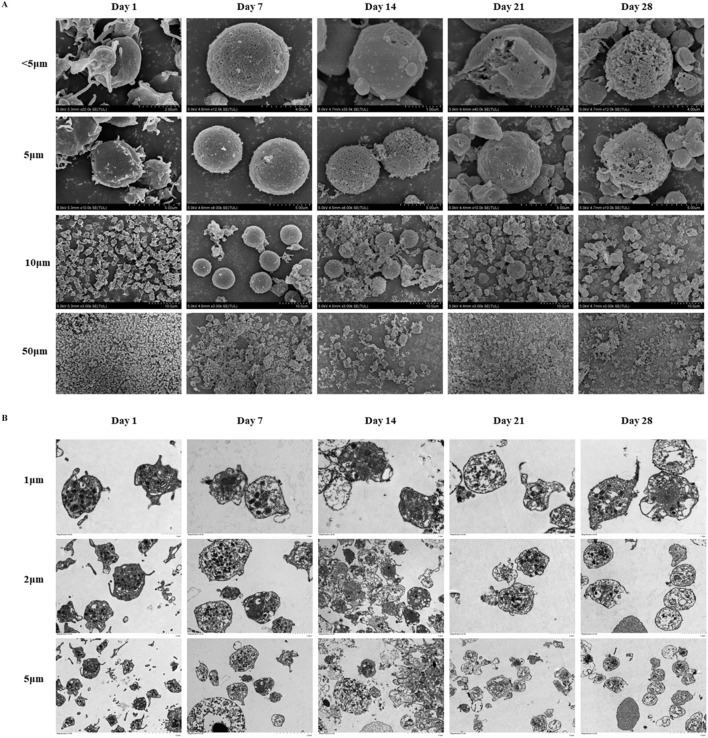
Platelets morphological changes in cold-stored whole blood (CS-WB). **(A)** Platelets morphological changes by scanning electron microscopy. Day 0: platelets displayed normal morphology with visible pseudopodia; Day 7: pseudopodia were nearly absent, and small platelets began to aggregate and adhere; Day 14: surface irregularities became more pronounced, indentations enlarge, and large platelets aggregates formed; Day 21: surface irregularities increased further, indentations expanded, and extensive platelet aggregation and adhesion were observed; Day 28: platelets lose their normal shape, and aggregation and adhesion were significantly more prominent. Day 35: no intact platelets were recoverable, and imaging was not possible, indicating severe degradation. **(B)** Platelets morphological changes by transmission electron microscope. Day 0: Platelets membrane structure was clear, and internal contents were intact; Day 7: internal contents became blurred, with a few small vacuoles appearing; Day 14: platelets membrane began to dissolve, and contents were released; Day 21: extensive dissolution of internal structures occurred within platelets; Day 28: dissolution of platelets contents intensifies, with numerous vacuoles present, indicating further degradation; Day 35: no intact platelets were recoverable, and imaging was not possible, indicating severe degradation.

The SEM observation results revealed distinct morphological changes as storage duration increased (Figure 4A). At the outset, platelets exhibited smooth surfaces with clear, round or oval outlines, and displayed pseudopod-like protrusions and minor indentations. Over time, these surface features loosened, pseudopods gradually disappeared, and pronounced indentations emerged, along with extensive platelet aggregation and adhesion. The TEM observation data showed that the platelet membrane was initially well-defined, and internal contents such as α particles, dense particles, and lysosomal particles appeared clear and intact (Figure 4B). However, the membrane integrity began to deteriorate, and intracellular components were released, with signifying advanced structural damage on day 14.

## 4 Discussion

Since the 1980s, widespread use of blood components in clinical practice has gradually reduced the use of WB. Some scholars believe that certain factors in WB become unstable as storage time increases. Additionally, platelets biological activity declines, limiting its clinical applicability. In pre-hospital emergency care and battlefield rescue, severe blood loss is a leading cause of death among trauma patients. Timely blood transfusion is crucial for survival. However, specific blood component products may be difficult to obtain simultaneously due to site and storage equipment limitations. WB product is able to provide all blood components simultaneously and is easy to store and transport, making it increasingly widely used for emergency treatment of trauma bleeding patients. RBCs, white blood cells, platelets, and plasma in WB interact in ways that can enhance buffering capacity and slow platelet metabolism (Huish et al., 2019; Hervig et al., 2021). Therefore, the shelf life of WB should not be determined solely by RBC viability. Instead, interactions among these components must also be considered to define its optimal usage period for trauma treatment.

RBCs are the primary blood components in the human body and play a crucial role in maintaining life. In this study, we analyzed the storage damage changes of RBCs components in WB product. The *in vitro* preservation results revealed that both structure and function of RBCs changed accompanied by prolonged storage time of WB. These changes affect the oxygen affinity of RBCs and their ability to transport oxygen in the circulatory system. Throughout the storage period, the basic physical and chemical properties of RBCs remained stable. However, a significant number of echinocytes appeared, and almost all RBCs lost their normal morphology on 28th day of refrigerated storage. The MCV of RBCs increased significantly, while the surface area remained unchanged. These changes resulted in reduced deformability and increased osmotic fragility, which accelerates RBC destruction and leads to the release of hemoglobin into the plasma and an increase in FHb concentration. An increase in FHb always increases the consumption of nitric oxide (NO) then leads to vasoconstriction, which may be unfavorable for emergency treatment of severely traumatized blood transfusion patients (Schechter and Gladwin, 2003; Sonveaux et al., 2007). Such deformities in RBCs may stem from declining levels of 2,3-DPG and increased externalization of PS. Our findings indicated that intracellular 2,3-DPG levels decreased significantly on day 14 of storage. As an allosteric effector of Hb, 2,3-DPG not only stabilizes the spatial conformation of Hb but also facilitates its oxygen release (Lang et al., 2003). The previous studies have shown that the externalization rate of PS increases significantly after 24 days of RBC storage (Stewart et al., 2005). In contrast, our findings indicate that a significant difference emerged only after 35 days, suggesting inconsistency between the two studies. Furthermore, elevated PS exposure suggests a loss of cytoskeletal stability, a key factor in echinocyte formation.

During *in vitro* storage, glycolysis continuously consumes glucose to produce lactic acid, leading to a decrease in pH within the blood bag. As glucose is consumed, the rate of glycolysis decreases, leading to reduced ATP production. This, in turn, compromises Na^+^-K^+^ pump function and exacerbates ion imbalance. K^+^ ions are released from inside the cells when RBCs are damaged, resulting in an increase in extracellular K^+^ concentration. Although it remains within the normal limits of blood quality standards, the concentration rises 4.81-fold by the end of storage. Raza S.et al. shows that transfusing RBC concentrates stored for more than 12 days can evidently raise blood K^+^ levels in patients (Raza et al., 2015). Most existing work only focuses on *in vitro* changes, with limited data on the *in vivo* effects of WB transfusion on K^+^ balance. Future studies should incorporate clinical transfusion data to evaluate the real-world risks that blood storage poses to human health especially for emergency blood transfusion for trauma patients.

For trauma-induced blood loss, it is essential not only to ensure oxygen supply and maintain circulating blood volume but also to correct trauma-related coagulopathies promptly. We examined multiple blood coagulation indicators in detail, to evaluate the coagulation function of WB product at different storage time points. The results showed that the activities of unstable clotting factors and stable clotting factors maintained more than 50% of their initial levels after 2 weeks. Beyond individual factor assays, monitoring of APTT, PT, and TEG-R values provided an integrated view of clotting factor performance. Both APTT and PT began to show clinically significant abnormal values after 14 days of storage. However, both Fib and TT stayed within normal ranges throughout the storage period, suggesting WB transfusion can provide sufficient Fib levels. TEG detects WB sample and reflects the interactions among various components in blood coagulation and fibrinolysis. It provides effective verification alongside conventional coagulation tests. R value primarily reflects the activity of coagulation factors. K value and Angle value exhibit the combined effect of Fib and platelets in blood clot formation (mainly based on Fib function). Our results showed that R value and Fib value began to appear significant changes after 28 days of refrigerated storage, while K value and Angle value began to present significant changes on storage day 21. Accordingly, we consider that CS-WB product can maintain a basic normal coagulation function after being stored for about 21 days.

Platelets are traditionally stored at room temperature with agitation. However, accumulating evidence supports refrigerated storage, citing benefits such as lower bacterial proliferation and prolonged shelf life (Ketter et al., 2019; Mack et al., 2020; Zhao and Devine, 2022). Cold-stored platelets effectively correct bleeding time within 24 h of transfusion and have a longer circulation time. Their safety and efficacy are comparable to those of room temperature-stored platelets (Valeri, 1976; Mack et al., 2020; Lu et al., 2023; Fisher et al., 2024). Recently, a multicenter phase-II randomized controlled trial demonstrated the operational feasibility of transfusing severely injured patients with a single cold-stored platelet unit (stored for 1–14 days). This approach reduces the time to first platelet transfusion and results in similar 24-h mortality rates (5.9% vs. 10.2%) compared to standard care, without increasing thromboembolic risks (Sperry et al., 2024). In urgent hemostasis, transfusion of cold platelets can serve as an effective alternative treatment. In the present study, we deeply explored the *in vitro* functional and morphological changes of platelets in CS-WB. The results showed that the platelet count in WB gradually decreased with increasing storage time and remained at 77.11% of the initial value on the 14th day. TEG-MA value reflects the maximum strength and stability of forming a blood clot, and it is mainly used to evaluate the role of platelets, including quantity, quality, and function. We found that MA value remained close to normal range within 3 weeks of storage, suggesting overall well-preserved platelet integrity and function.

In the early stages of storage, platelets energy metabolism primarily relies on the oxygen present in the plasma for aerobic respiration. However, oxygen levels gradually decrease as storage time progresses. Consequently, platelets rely more on anaerobic glycolysis, causing the accumulation of byproducts such as lactate and hydrogen ions (D'Alessandro et al., 2020; Liu et al., 2024). These changes further result in platelet damage, which compromises their viability and hemostatic function and reduces the hemostatic effectiveness of blood transfusion in recipients. On day 7 of storage, PS exposure on the platelet surface and CD62P expression increase significantly, indicating a gradual rise in platelet apoptosis and activation. Specifically, PS exposure results from the outward flipping of the platelet membrane’s inner leaflet, while CD62P arises from the release of platelet internal granules. The outward flipping of PS and the release of granular substances are closely related to mitochondrial membrane potential damage, reduced glucose levels due to continuous metabolic activity, and the apoptosis mechanism triggered by lactate accumulation (Bertino et al., 2003; Albanyan et al., 2009).

The observation results from SEM and TEM further demonstrated that platelets surfaces were dense and well-defined and featured pseudopod protrusions in the early phase of WB storage. After storage for 14 days, significant changes in platelets morphology were observed. The surface structure became looser, the membrane integrity was disrupted, and pseudopods disappeared, with large areas of aggregation and adhesion forming. At the molecular level, the expression of G-protein-coupled enzyme-activated receptors decreases, which is associated with the exposure and shedding of CD42b, GPVI, and PS (Dasgupta et al., 2010; Hosseini et al., 2017; Vucetic et al., 2018). Additionally, platelets modulate T cell activation and affect their interactions with the extracellular environment. This occurs through the translocation of CD62P from α-granules to the cell surface and the secretion of soluble CD40L (Wakamoto et al., 2003; Sahler et al., 2012). Our findings suggest that CS-WB generally maintains relatively normal platelets function and morphology for up to 14 days. Nevertheless, further clinical studies are required to determine appropriate storage period of WB product for treatment of trauma bleeding patients *in vivo*.

Several limitations in our study merit consideration. First, WB products were stored statically at 4 °C without agitation, but minor agitation effects cannot be completely excluded. Second, repeated sampling removed approximately 24% of the original volume, however, this volume reduction may alter storage dynamics. These factors should nevertheless be acknowledged when extrapolating our results.

Future studies should focus on comprehensive transfusion medicine guidelines and storage standards. Although CPDA remains the anticoagulant of choice in military blood banking, recent studies indicate that novel nutrient-enriched preservative solutions could better maintain ATP levels, platelet count, and hemostatic function during refrigerated storage (Reddoch-Cardenas et al., 2023; Reddoch-Cardenas et al., 2025). Head-to-head comparisons of CPDA and fortified formulations are warranted to clarify whether enhanced metabolic support can significantly delay storage lesions. Additionally, while leukocyte reduction provides important safety benefits, its impact on platelet function and early hemostatic capacity remains to be fully elucidated. Therefore, future research should directly compare standard and platelet-sparing LR methods under identical cold-storage conditions, aiming to identify an optimal balance between transfusion safety and functional preservation.

## 5 Conclusion

Although WB is increasingly recognized as an essential component of damage control resuscitation, the studies on its storage lesion *in vitro* and *in vivo* effects remains relatively sparse. Based on our limited *in vitro* data, we speculate that CS-WB stored for up to 14 days preserves the oxygen-carrying capacity of RBCs and retains the hemostatic function of coagulation factors and platelets at relatively high levels. Moreover, CS-WB stored for up to 21 days shows relatively low RBC damage and maintains effective plasma coagulation function but reduced platelets hemostatic ability. Therefore, we infer that CS-WB stored for up to 14 days offers significant advantages in maintaining normal oxygen transport, coagulation, and hemostatic functions. For patients with hemorrhagic shock due to trauma, such CS-WB is particularly beneficial during resuscitation, especially in emergency or wartime scenarios, or in pre-hospital care settings where immediate access to specialized blood products may be limited.

## Data Availability

The datasets presented in this article are not readily available because The data that support the findings of this study are available on request from the corresponding author, upon reasonable request. Requests to access the datasets should be directed to Lei Liu, liulei890207@163.com.

## Glossary

WB: Whole blood
CS-WB: Cold-stored whole blood
RBCs: Red blood cells
CPDA: Citrate phosphate dextrose adenine
TEG: Thromboelastograph
NaCl: Sodium chloride
ELISA: Enzyme-Linked Immunosorbent Assay
PS: Phosphatidylserine
FHb: Free hemoglobin
2,3-DPG: 2,3-diphosphoglycerate
APTT: Activated partial thromboplastin time
PT: Prothrombin time
TT: Thrombin time
Fib: Fibrinogen
FVIII: Coagulation factor VIII
ALB: Albumin
GLB: Globulin
**CO**_**2**_: Carbon dioxide
Lac: Lactate
**K** ^**+**^: Potassium ion
**Na** ^**+**^: Sodium ion
**Ca** ^**2+**^: Calcium ion
GLU: Glucose
LDH: Lactic dehydrogenase
SEM: Scanning electron microscopy
TEM: Transmission electron microscopy
SPSS: Statistical Package for the Social Sciences
SD: Standard deviations
ANOVA: One-way analysis of variance
Hct: Hematocrit
Hb: Hemoglobin
MCV: Mean corpuscular volume
MPV: Mean platelet volume
PDW: Platelet distribution width
NO: Nitric oxide
