# Supplementary material for: Storage damage of cold-stored whole blood in CPDA maintenance fluid during the whole effective storage period
Source: Front Cell Dev Biol. 2025 Aug 22;13:1610009. doi: 10.3389/fcell.2025.1610009 (PMC12411527; doi:10.3389/fcell.2025.1610009)
Supplement: Supplementary file 1 [file Supplementaryfile1.docx]

**Supplementary Table 1. Pair-wise comparisons between baseline (Day 0) and the five subsequent storage days across 38 laboratory indicators**

| **Indicators** | **Contrast (Baseline vs.)** | **Mean Diff.** | **95 % CI of Diff. †** | **Raw *P*** | **Adj. *P* (BH) ‡** |
| --- | --- | --- | --- | --- | --- |
| RBC (*10^12^/L) | Day 7 | -0.04 | -0.32 to 0.23 | 0.997 | 1.000 |
|  | Day 14 | 0.04 | -0.25 to 0.34 | 0.997 | 1.000 |
|  | Day 21 | 0.03 | -0.25 to 0.30 | 1.000 | 1.000 |
|  | Day 28 | -0.03 | -0.31 to 0.24 | 0.999 | 1.000 |
|  | Day 35 | 0.10 | -0.17 to 0.38 | 0.869 | 1.000 |
| Hb (g/L) | Day 7 | -0.65 | -8.82 to 7.52 | 1.000 | 1.000 |
|  | Day 14 | 1.83 | -6.84 to 10.49 | 0.987 | 1.000 |
|  | Day 21 | 0.40 | -7.77 to 8.57 | 1.000 | 1.000 |
|  | Day 28 | -0.10 | -8.27 to 8.07 | 1.000 | 1.000 |
|  | Day 35 | 3.50 | -4.67 to 11.67 | 0.786 | 1.000 |
|  | Day 7 | -1.15 | -3.64 to 1.35 | 0.735 | 1.000 |
|  | Day 14 | -0.84 | -3.49 to 1.81 | 0.927 | 1.000 |
|  | Day 21 | -0.73 | -3.23 to 1.77 | 0.948 | 1.000 |
|  | Day 28 | -1.68 | -4.17 to 0.82 | 0.350 | 0.523 |
|  | Day 35 | -0.47 | -2.97 to 2.03 | 0.993 | 1.000 |
| MCV (fL) | Day 7 | -1.73 | -4.76 to 1.31 | 0.531 | 0.764 |
|  | Day 14 | -2.01 | -5.05 to 1.03 | 0.364 | 0.540 |
|  | Day 21 | -2.35 | -5.38 to 0.69 | 0.210 | 0.321 |
|  | Day 28 | -3.24 | -6.27 to -0.20 | 0.031 | 0.055 |
|  | Day 35 | -3.53 | -6.57 to -0.49 | 0.015 | 0.028 |
| EC_50_  (%) | Day 7 | -9.47 | -18.98 to 0.05 | 0.052 | 0.086 |
|  | Day 14 | -13.17 | -22.35 to -4.00 | 0.002 | 0.003 |
|  | Day 21 | -13.97 | -23.48 to -4.46 | 0.001 | 0.003 |
|  | Day 28 | -13.38 | -22.71 to -4.04 | 0.002 | 0.003 |
|  | Day 35 | -14.83 | -23.73 to -5.93 | <0.001 | 0.001 |
| FHb (μmol/g) | Day 7 | -0.01 | -0.12 to 0.09 | 0.999 | 1.000 |
|  | Day 14 | -0.04 | -0.15 to 0.06 | 0.814 | 1.000 |
|  | Day 21 | -0.09 | -0.19 to 0.01 | 0.105 | 0.168 |
|  | Day 28 | -0.24 | -0.35 to -0.14 | <0.001 | <0.001 |
|  | Day 35 | -0.37 | -0.48 to -0.26 | <0.001 | <0.001 |
| PS  (%) | Day 7 | -0.30 | -2.31 to 1.71 | 0.997 | 1.000 |
|  | Day 14 | -0.28 | -2.23 to 1.68 | 0.998 | 1.000 |
|  | Day 21 | -1.43 | -3.38 to 0.53 | 0.264 | 0.401 |
|  | Day 28 | -2.07 | -4.03 to -0.11 | 0.033 | 0.057 |
|  | Day 35 | -4.66 | -6.62 to -2.71 | <0.001 | <0.001 |
| 2，3-DPG (nmol/mL) | Day 7 | 0.92 | -12.91 to 14.74 | 1.000 | 1.000 |
|  | Day 14 | 15.17 | 1.52 to 28.81 | 0.022 | 0.040 |
|  | Day 21 | 17.48 | 3.84 to 31.12 | 0.006 | 0.011 |
|  | Day 28 | 17.14 | 3.50 to 30.79 | 0.007 | 0.014 |
|  | Day 35 | 19.12 | 5.47 to 32.76 | 0.002 | 0.004 |
| ATP (μmol/g Hb) | Day 7 | 1.50 | -0.32 to 3.31 | 0.158 | 0.248 |
|  | Day 14 | 3.36 | 1.61 to 5.10 | <0.001 | <0.001 |
|  | Day 21 | 3.97 | 2.23 to 5.72 | <0.001 | <0.001 |
|  | Day 28 | 4.00 | 2.19 to 5.82 | <0.001 | <0.001 |
|  | Day 35 | 4.08 | 2.26 to 5.89 | <0.001 | <0.001 |
| EMA (%) | Day 7 | 8.35 | 1.25 to 15.45 | 0.014 | 0.026 |
|  | Day 14 | 12.74 | 6.35 to 19.14 | <0.001 | <0.001 |
|  | Day 21 | 13.99 | 6.90 to 21.09 | <0.001 | <0.001 |
|  | Day 28 | 16.84 | 10.45 to 23.24 | <0.001 | <0.001 |
|  | Day 35 | 17.85 | 11.45 to 24.24 | <0.001 | <0.001 |
| APTT (S) | Day 7 | -4.62 | -9.20 to -0.03 | 0.048 | 0.081 |
|  | Day 14 | -6.06 | -10.71 to -1.41 | 0.006 | 0.011 |
|  | Day 21 | -12.20 | -16.79 to -7.61 | <0.001 | <0.001 |
|  | Day 28 | -13.32 | -17.90 to -8.73 | <0.001 | <0.001 |
|  | Day 35 | -15.06 | -19.84 to -10.28 | <0.001 | <0.001 |
| PT  (S) | Day 7 | -1.23 | -2.26 to -0.19 | 0.012 | 0.024 |
|  | Day 14 | -2.17 | -3.20 to -1.13 | <0.001 | <0.001 |
|  | Day 21 | -2.97 | -4.00 to -1.93 | <0.001 | <0.001 |
|  | Day 28 | -3.83 | -4.86 to -2.79 | <0.001 | <0.001 |
|  | Day 35 | -4.29 | -5.32 to -3.25 | <0.001 | <0.001 |
| TT  (S) | Day 7 | 0.34 | -0.54 to 1.22 | 0.845 | 1.000 |
|  | Day 14 | -0.98 | -1.86 to -0.10 | 0.021 | 0.039 |
|  | Day 21 | -1.30 | -2.18 to -0.42 | 0.001 | 0.002 |
|  | Day 28 | -2.74 | -3.62 to -1.86 | <0.001 | <0.001 |
|  | Day 35 | -3.18 | -4.06 to -2.30 | <0.001 | <0.001 |
| Fib  (g/L) | Day 7 | 0.10 | -0.30 to 0.50 | 0.974 | 1.000 |
|  | Day 14 | 0.24 | -0.17 to 0.64 | 0.499 | 0.730 |
|  | Day 21 | 0.36 | -0.04 to 0.77 | 0.096 | 0.156 |
|  | Day 28 | 0.43 | 0.03 to 0.84 | 0.029 | 0.052 |
|  | Day 35 | 0.57 | 0.16 to 0.97 | 0.002 | 0.004 |
| K  (min) | Day 7 | -0.13 | -0.77 to 0.52 | 0.991 | 1.000 |
|  | Day 14 | -0.27 | -0.93 to 0.39 | 0.819 | 1.000 |
|  | Day 21 | -0.87 | -1.51 to -0.23 | 0.003 | 0.006 |
|  | Day 28 | -1.67 | -2.32 to -1.02 | <0.001 | <0.001 |
|  | Day 35 | -2.64 | -3.28 to -2.00 | <0.001 | <0.001 |
| Angel  (°) | Day 7 | 0.86 | -3.53 to 5.25 | 0.991 | 1.000 |
|  | Day 14 | 3.63 | -0.88 to 8.14 | 0.174 | 0.271 |
|  | Day 21 | 7.03 | 2.64 to 11.41 | <0.001 | 0.001 |
|  | Day 28 | 12.78 | 8.39 to 17.17 | <0.001 | <0.001 |
|  | Day 35 | 14.57 | 10.18 to 18.95 | <0.001 | <0.001 |
| R  (min) | Day 7 | -0.26 | -0.95 to 0.43 | 0.873 | 1.000 |
|  | Day 14 | -0.75 | -1.49 to -0.02 | 0.051 | 0.086 |
|  | Day 21 | -0.64 | -1.34 to 0.05 | 0.098 | 0.158 |
|  | Day 28 | -0.82 | -1.51 to -0.14 | 0.015 | 0.028 |
|  | Day 35 | -1.02 | -1.71 to -0.34 | 0.001 | 0.003 |
| FⅧ  (%) | Day 7 | 18.18 | 3.67 to 32.70 | 0.007 | 0.014 |
|  | Day 14 | 22.66 | 7.96 to 37.36 | 0.001 | 0.001 |
|  | Day 21 | 29.82 | 15.31 to 44.34 | <0.001 | <0.001 |
|  | Day 28 | 33.37 | 18.86 to 47.89 | <0.001 | <0.001 |
|  | Day 35 | 38.02 | 23.51 to 52.54 | <0.001 | <0.001 |
| FⅩⅢ（pg/mL） | Day 7 | 4.25 | -10.42 to 18.92 | 0.950 | 1.000 |
|  | Day 14 | 11.65 | -3.21 to 26.51 | 0.197 | 0.304 |
|  | Day 21 | 21.30 | 6.63 to 35.97 | 0.001 | 0.003 |
|  | Day 28 | 22.34 | 7.67 to 37.01 | 0.001 | 0.001 |
|  | Day 35 | 29.35 | 14.68 to 44.02 | <0.001 | <0.001 |
| FⅩ（pg/mL） | Day 7 | 7.73 | -9.04 to 24.49 | 0.731 | 1.000 |
|  | Day 14 | 21.90 | 5.14 to 38.66 | 0.005 | 0.009 |
|  | Day 21 | 24.21 | 7.45 to 40.97 | 0.001 | 0.003 |
|  | Day 28 | 23.87 | 7.11 to 40.64 | 0.002 | 0.003 |
|  | Day 35 | 25.85 | 9.09 to 42.61 | 0.001 | 0.001 |
| PLT (*109/L) | Day 7 | 19.70 | -7.94 to 47.34 | 0.286 | 0.431 |
|  | Day 14 | 38.40 | 10.76 to 66.04 | 0.002 | 0.004 |
|  | Day 21 | 44.55 | 16.91 to 72.19 | <0.001 | 0.001 |
|  | Day 28 | 48.35 | 20.71 to 75.99 | <0.001 | <0.001 |
|  | Day 35 | 47.59 | 19.20 to 75.99 | <0.001 | <0.001 |
| PDW  (fL) | Day 7 | 0.45 | -2.36 to 3.26 | 0.997 | 1.000 |
|  | Day 14 | 0.91 | -2.03 to 3.84 | 0.935 | 1.000 |
|  | Day 21 | 0.28 | -2.57 to 3.12 | 1.000 | 1.000 |
|  | Day 28 | 0.12 | -2.65 to 2.89 | 1.000 | 1.000 |
|  | Day 35 | 0.37 | -2.41 to 3.14 | 0.999 | 1.000 |
| MPV  (fL) | Day 7 | 0.10 | -0.96 to 1.16 | 1.000 | 1.000 |
|  | Day 14 | 0.21 | -0.86 to 1.27 | 0.992 | 1.000 |
|  | Day 21 | 0.27 | -0.79 to 1.33 | 0.971 | 1.000 |
|  | Day 28 | 0.38 | -0.69 to 1.44 | 0.891 | 1.000 |
|  | Day 35 | 0.49 | -0.57 to 1.55 | 0.729 | 1.000 |
| MA  (mm) | Day 7 | 0.88 | -3.00 to 4.75 | 0.983 | 1.000 |
|  | Day 14 | 1.11 | -2.87 to 5.09 | 0.957 | 1.000 |
|  | Day 21 | 6.41 | 2.53 to 10.28 | <0.001 | <0.001 |
|  | Day 28 | 11.89 | 8.01 to 15.77 | <0.001 | <0.001 |
|  | Day 35 | 17.04 | 13.16 to 20.91 | <0.001 | <0.001 |
| CD62P (%) | Day 7 | -9.88 | -16.05 to -3.71 | 0.000 | 0.001 |
|  | Day 14 | -19.75 | -25.85 to -13.65 | <0.001 | <0.001 |
|  | Day 21 | -17.70 | -23.80 to -11.60 | <0.001 | <0.001 |
|  | Day 28 | -16.08 | -22.18 to -9.98 | <0.001 | <0.001 |
|  | Day 35 | -12.43 | -18.60 to -6.26 | <0.001 | <0.001 |
| PS  (%) | Day 7 | -4.23 | -11.68 to 3.22 | 0.531 | 0.764 |
|  | Day 14 | -18.12 | -25.57 to -10.67 | <0.001 | <0.001 |
|  | Day 21 | -31.33 | -38.87 to -23.79 | <0.001 | <0.001 |
|  | Day 28 | -36.69 | -44.13 to -29.24 | <0.001 | <0.001 |
|  | Day 35 | -44.95 | -52.39 to -37.50 | <0.001 | <0.001 |
| MCH  (pg) | Day 7 | 0.14 | -0.63 to 0.91 | 0.993 | 1.000 |
|  | Day 14 | 0.21 | -0.56 to 0.97 | 0.964 | 1.000 |
|  | Day 21 | -0.11 | -0.88 to 0.66 | 0.998 | 1.000 |
|  | Day 28 | 0.19 | -0.58 to 0.96 | 0.974 | 1.000 |
|  | Day 35 | 0.09 | -0.68 to 0.85 | 0.999 | 1.000 |
| MCHC (g/L) | Day 7 | 7.15 | -0.55 to 14.85 | 0.082 | 0.134 |
|  | Day 14 | 8.35 | 0.65 to 16.05 | 0.027 | 0.049 |
|  | Day 21 | 6.45 | -1.25 to 14.15 | 0.145 | 0.229 |
|  | Day 28 | 12.20 | 4.50 to 19.90 | <0.001 | 0.001 |
|  | Day 35 | 12.00 | 4.30 to 19.70 | <0.001 | 0.001 |
| WBC (*109/L) | Day 7 | 0.79 | 0.04 to 1.53 | 0.033 | 0.057 |
|  | Day 14 | 1.36 | 0.61 to 2.10 | <0.001 | <0.001 |
|  | Day 21 | 1.73 | 0.99 to 2.47 | <0.001 | <0.001 |
|  | Day 28 | 2.04 | 1.29 to 2.78 | <0.001 | <0.001 |
|  | Day 35 | 2.16 | 1.42 to 2.90 | <0.001 | <0.001 |
| ALB  (g/L) | Day 7 | -0.23 | -1.49 to 1.04 | 0.994 | 1.000 |
|  | Day 14 | -0.02 | -1.29 to 1.25 | 1.000 | 1.000 |
|  | Day 21 | -0.23 | -1.50 to 1.04 | 0.994 | 1.000 |
|  | Day 28 | 0.20 | -1.07 to 1.46 | 0.997 | 1.000 |
|  | Day 35 | -1.32 | -2.58 to -0.05 | 0.038 | 0.066 |
| GLOB (mmol/L) | Day 7 | -0.83 | -2.95 to 1.30 | 0.845 | 1.000 |
|  | Day 14 | -0.83 | -3.11 to 1.46 | 0.882 | 1.000 |
|  | Day 21 | -1.04 | -3.16 to 1.09 | 0.681 | 0.966 |
|  | Day 28 | -1.01 | -3.13 to 1.11 | 0.703 | 0.989 |
|  | Day 35 | -0.92 | -3.04 to 1.20 | 0.777 | 1.000 |
| CO_2_ (mmol/L) | Day 7 | 1.39 | -0.09 to 2.87 | 0.075 | 0.124 |
|  | Day 14 | 3.34 | 1.86 to 4.82 | <0.001 | <0.001 |
|  | Day 21 | 4.93 | 3.45 to 6.40 | <0.001 | <0.001 |
|  | Day 28 | 6.40 | 4.92 to 7.88 | <0.001 | <0.001 |
|  | Day 35 | 9.34 | 7.86 to 10.81 | <0.001 | <0.001 |
| Lac (mmol/L) | Day 7 | -2.41 | -3.10 to -1.71 | <0.001 | <0.001 |
|  | Day 14 | -3.89 | -4.59 to -3.20 | <0.001 | <0.001 |
|  | Day 21 | -5.22 | -5.91 to -4.52 | <0.001 | <0.001 |
|  | Day 28 | -7.13 | -7.83 to -6.44 | <0.001 | <0.001 |
|  | Day 35 | -6.95 | -7.64 to -6.25 | <0.001 | <0.001 |
| K+ (mmol/L) | Day 7 | -6.51 | -8.16 to -4.86 | <0.001 | <0.001 |
|  | Day 14 | -9.72 | -11.47 to -7.97 | <0.001 | <0.001 |
|  | Day 21 | -15.10 | -16.75 to -13.45 | <0.001 | <0.001 |
|  | Day 28 | -19.17 | -20.82 to -17.52 | <0.001 | <0.001 |
|  | Day 35 | -21.24 | -22.89 to -19.59 | <0.001 | <0.001 |
| Na+ (mmol/L) | Day 7 | 3.52 | 1.39 to 5.65 | <0.001 | <0.001 |
|  | Day 14 | 4.81 | 2.68 to 6.94 | <0.001 | <0.001 |
|  | Day 21 | 9.18 | 7.05 to 11.30 | <0.001 | <0.001 |
|  | Day 28 | 13.44 | 11.29 to 15.59 | <0.001 | <0.001 |
|  | Day 35 | 13.40 | 11.05 to 15.74 | <0.001 | <0.001 |
| Ca^2+^ (mmol/L) | Day 7 | 0.04 | -0.06 to 0.13 | 0.831 | 1.000 |
|  | Day 14 | -0.03 | -0.12 to 0.07 | 0.934 | 1.000 |
|  | Day 21 | 0.04 | -0.05 to 0.14 | 0.728 | 1.000 |
|  | Day 28 | 0.04 | -0.05 to 0.14 | 0.756 | 1.000 |
|  | Day 35 | -0.33 | -0.42 to -0.23 | <0.001 | <0.001 |
| Glu (mmol/L) | Day 7 | 3.20 | -2.58 to 8.97 | 0.560 | 0.799 |
|  | Day 14 | 5.83 | 0.05 to 11.61 | 0.047 | 0.080 |
|  | Day 21 | 7.64 | 1.86 to 13.42 | 0.004 | 0.008 |
|  | Day 28 | 10.42 | 4.64 to 16.19 | <0.001 | <0.001 |
|  | Day 35 | 10.98 | 5.20 to 16.75 | <0.001 | <0.001 |
| LDH (mmol/L) | Day 7 | -65.00 | -171.00 to 41.05 | 0.448 | 0.660 |
|  | Day 14 | -137.90 | -243.90 to -31.80 | 0.005 | 0.010 |
|  | Day 21 | -256.00 | -362.00 to -149.90 | <0.001 | <0.001 |
|  | Day 28 | -366.10 | -472.10 to -260.10 | <0.001 | <0.001 |
|  | Day 35 | -523.10 | -629.10 to -417.10 | <0.001 | <0.001 |

† Mean Diff. = Mean_DayX_ − Mean_Day0_. Positive values indicate an increase relative to baseline. Units follow those of each indicator.
‡ Adjusted p values were obtained with the Benjamini–Hochberg procedure, controlling the false discovery rate at 5 % across all 190 planned contrasts (38 indicators × 5 comparisons).
CI = confidence interval; Laboratory abbreviations are detailed in the Abbreviations list.

**Supplemental Table 2. Trends in haemostatic metrics during the storage period of whole blood**

| **Haemostatic metrics** | **Day 0** | **Day 7** | **Day 14** | **Day 21** | **Day 28** | **Day 35** |
| --- | --- | --- | --- | --- | --- | --- |
| PLT  (*10^9^/L) | 184.70 ± 37.16 | 165.00 ± 40.55 | 146.30 ± 36.87* | 140.10 ± 26.61* | 136.30 ± 30.60* | 137.10 ± 25.14* |
| R  (min) | 5.72± 0.84 | 5.98 ± 0.88 | 6.47 ± 0.64 | 6.36 ± 0.61 | 6.54 ± 0.69* | 6.7 ± 0.96* |
| K  (min) | 1.87 ± 0.36 | 1.99 ± 0.31 | 2.13 ± 0.33 | 2.74 ± 0.53* | 3.53 ± 0.83* | 4.51 ± 1.51* |
| Angle  (°) | 64.15 ± 3.96 | 63.29 ± 3.29 | 60.52 ± 4.09 | 57.13 ± 4.71* | 51.37 ± 6.56* | 49.59 ± 7.72* |
| MA  (mm) | 59.74 ± 3.06 | 58.87 ± 3.31 | 58.63 ± 3.62 | 53.34 ± 4.25* | 47.85 ± 5.96* | 42.71 ± 6.64* |
| Fib  (g/L) | 3.06 ± 0.49 | 2.96 ± 0.53 | 2.83 ± 0.49 | 2.69 ± 0.49 | 2.63 ± 0.47* | 2.50 ± 0.47* |

* Indicates a statistically significant difference compared to the Day 0 group.

PLT=Platelet Count; R=Reaction Time; K=Clot Formation Time; MA= Maximum Amplitude; Fib= Fibrinogen
